# Recent advances in understanding RAG deficiencies

**DOI:** 10.12688/f1000research.17056.1

**Published:** 2019-02-04

**Authors:** Andrew Gennery

**Affiliations:** 1 Paediatric Immunology and Haematopoietic Stem Cell Transplantation, Great North Childrens’ Hospital, Newcastle upon Tyne, UK; 2 Primary Immunodeficiency Group, Institute of Cellular Medicine, Newcastle University, Newcastle upon Tyne, UK

**Keywords:** Atypical SCID; Autoimmunity; Omenn syndrome; Recombination-activating genes; Severe combined immunodeficiency (SCID); VDJ recombination;

## Abstract

Recombination-activating genes ( *RAG*) *1* and *RAG2 *initiate the molecular processes that lead to lymphocyte receptor formation through VDJ recombination. Nonsense mutations in *RAG1*/ *RAG2* cause the most profound immunodeficiency syndrome, severe combined immunodeficiency (SCID). Other severe and less-severe clinical phenotypes due to mutations in *RAG* genes are now recognized. The degree of residual protein function may permit some lymphocyte receptor formation, which confers a less-severe clinical phenotype. Many of the non-SCID phenotypes are associated with autoimmunity. New findings into the effect of mutations in *RAG1/2* on the developing T- and B-lymphocyte receptor give insight into the development of autoimmunity. This article summarizes recent findings and places the genetic and molecular findings in a clinical context.

## Introduction

Recombination-activating genes ( *RAG*) *1* and *RAG2* encode endonuclease proteins, which are critical to initiate the molecular processes that lead to lymphocyte receptor formation. Nonsense mutations in *RAG1*/ *RAG2* completely annul this process and abrogate T- and B-lymphocyte receptor formation, leading to the most profound immunodeficiency syndrome, severe combined immunodeficiency (SCID). A spectrum of less-severe clinical phenotypes is now recognized to be due to mutations in *RAG* genes; recent understanding of the impact of mutations on protein function helps to explain, at least some of, these features. This article summarizes recent findings and places the genetic and molecular findings in a clinical context.

## Clinical phenotypes of RAG deficiency

The recombination activity retained by RAG mutants correlates with the severity of clinical presentation, in that the least recombination activity is associated with the most-severe phenotype.

### Severe combined immunodeficiency

Nonsense mutations in *RAG1* or *RAG2* abolish the initiation of antigen receptor recombination, which prevents the progression of T- and B-lymphocyte progenitors beyond the DN3 and pre-B-1 stage of development, giving rise to a T-B-natural killer cell (NK)+ SCID phenotype ^1^. Infants classically present with infectious symptoms caused by common viral pathogens, which include respiratory viruses such as respiratory syncytial virus and parainfluenza viruses, as well as cytomegalovirus and adenoviruses and viruses causing enteritis, including rotavirus (which may be acquired from live-attenuated vaccine) and norovirus ^2^. Susceptibility to opportunistic pathogens such as *Pneumocystis jirovecii*, or attenuated vaccine organisms used in live vaccines, including BCG, and poliovirus vaccines is pathognomic ^2, 3^. Infections persist, causing pneumonitis, enteritis, and failure to thrive, and may disseminate.

### Omenn syndrome and ‘leaky’ severe combined immunodeficiency

Missense mutations in *RAG1* or *RAG2* severely disrupt the function of the recombinase proteins but permit occasional recombination events that maintain partial V(D)J recombination activity and lead to the expansion of oligoclonal T-lymphocyte populations ^4^. A study of RAG-deficient patients established that null mutations on both *RAG* alleles give rise to the T-B- SCID phenotype, whereas those patients who manifested classical Omenn syndrome harbored missense mutations on at least one *RAG* allele that permitted partial V(D)J recombination activity, which enabled the generation of residual, oligoclonal T lymphocytes ^5^. Patients with Omenn syndrome present with generalized lichenified protein-losing erythroderma, often associated with scaling and exfoliation. Scalp, and often eyebrow and eyelash, hair is lost with evolution of the rash; severe alopecia is characteristic and an important clinical indication of the diagnosis. The rash is usually present at birth or within a few days afterwards but may evolve over the first few weeks of life. Axillary and inguinal lymphadenopathy with hepatosplenomegaly is a frequent finding. Inflammatory pneumonitis, enteritis, or hepatitis may be present. Co-existing infection with conventional or opportunistic pathogens is usually demonstrated.

Immunological investigations reveal a T-lymphocytosis with a highly activated phenotype, dominated by a restricted oligoclonal expansion of a few TCRVβ families and absence of other families ^6– 8^. T-lymphocytes fail to proliferate in response to stimulation with phytohemagglutinin. Indicators of thymopoiesis and B-lymphocytes are absent and NK cells are generally present in normal numbers. Serum immunoglobulins IgM, IgA, and IgG are absent, with absence of vaccine antigen responses, but the serum IgE is usually raised, with an associated eosinophilia. A group of patients with a milder phenotype than classical Omenn syndrome, known as ‘leaky’ or ‘atypical’ SCID, were also shown to harbor missense mutations in *RAG1* or *RAG2* ^5^. Genotype is important in conferring immunotypes, as demonstrated by a patient with a classical nonsense 2113delC homozygous mutation in *RAG1* genomic DNA but who exhibited a typical clinico-hemato-immunophenotype associated with Omenn syndrome ^9^. The mutation was confirmed in both parents as heterozygote carriers and found in a homozygous state in genomic DNA from granulocytes from the patient. Contrary to predictions of a frameshift with abolition of protein function, DNA extracted from the patient’s peripheral CD4+ and CD8+ T-lymphocytes showed six co-existent somatic second-site missense mutations (absent from monocytes, granulocytes, and NK cells) along with the base C deletion. These compensatory mutations acted to restore the *RAG1* reading frame and suggested that the revertant somatic mutations occurred in early T-lymphocyte progenitors, demonstrating the importance of partial *RAG* function in the evolution of Omenn syndrome or atypical SCID. A similar case has since been reported ^10^. Other phenotypes associated with missense *RAG* mutations include patients who exhibit specific antibody production and autoantibody production despite profound B-lymphocytopenia and those who exhibit T-lymphocytopenia for TCRαβ-expressing lymphocytes but normal numbers of TCRγδ-expressing T-lymphocytes ^11^. A similar phenotype is described in which *RAG1* missense mutations were associated with autoimmune cytopenia and oligoclonal expansion of TCRγδ T-lymphocytes with TCRαβ+ T-lymphocytopenia ^12^ in the context of severe, disseminated CMV infection.

Not only is the division between SCID, atypical SCID, and Omenn syndrome dependent on gene mutation, demonstrated by the finding that siblings within a family can demonstrate alternate phenotypes ^13^, but also environmental factors can contribute to the phenotype. One study described three related patients presenting with classical immunological and phenotypic features of T-B-NK+ SCID, who harbored a homozygous mutation in the core domain of *RAG2* (1305G>T) ^14^, previously described in patients with T-B-NK+ SCID and in patients with Omenn syndrome ^15^. In the course of one month, a clinico-hemato-immunophenotype was seen to develop in the third patient, which was consistent with classical Omenn syndrome, following a parainfluenza type 3 lower respiratory tract infection ^14^. This case implies that environmental factors may act as a trigger for the evolution of a SCID phenotype into Omenn syndrome; in this case, an external infectious agent acted as a trigger, although other triggers could be unidentified internal stimuli.

### Combined immunodeficiency with granulomas

A syndrome characterized by later-onset infection with midline or widespread skin or deep-seated granulomatous lesions is now well described ^16– 18^. Severe viral or vaccine-induced infection is described, with fatal sepsis in childhood. Autoimmune features are also described. Immunoglobulin levels, vaccine responses, and lymphocyte numbers are variably absent, low, or normal. Missense mutations in *RAG* have been described in affected patients. A further adult patient who harbored a frameshift and a missense mutation with reduced function in *RAG1* has been described with combined immunodeficiency, who died from JC virus-associated progressive multifocal leukoencephalopathy ^19^. Granulomas identified in RAG-deficient patients generally have no identifiable infectious or systemic cause, but vaccine strain rubella virus can create chronic infection in M2 macrophages and keratinocytes within granulomas of patients with various T-lymphocyte defects, including RAG deficiency, suggesting a role for chronic viral infection and immune dysfunction in granuloma formation ^20^.

### Common variable immunodeficiency

Common variable immunodeficiency (CVID), typically associated with hypogammaglobulinemia and low or nonexistent specific antibody responses to vaccination, comprises a heterogeneous group of primary immunodeficiencies. Other non-infectious features are common, including autoimmune cytopenias, granulomatous disease, and enteropathy. Around 10% of patients have a genetic diagnosis, and, in some, missense *RAG* mutations have been implicated ^21, 22^.

### Antibody deficiency

Although most cases of *RAG* deficiency described to date associate with a greater or lesser degree of T-lymphocyte deficiency, isolated agammaglobulinemia is described ^23, 24^, as well as polysaccharide antibody deficiency in adults ^25^, and a patient with isolated IgA deficiency is also described ^26^ with associated missense mutations in *RAG*.

### Autoimmunity

Infectious manifestations are the predominant presenting feature in *RAG* deficiency, but autoimmune features are the predominant presenting phenotype in a number of patients, including hematological cytopenias, autoimmune hepatitis, myopathy, and nephrotic syndrome, with associated *RAG* missense mutations ^27, 28^. Intriguingly, one patient developed systemic lupus erythematosus with erosive arthritis and was demonstrated to have a heterozygous *RAG2* missense mutation associated with diminished lymphocyte receptor editing ^29^.

### Miscellaneous presentations

A number of other immunological presentations are described, including isolated idiopathic CD4+ lymphocytopenia ^30^, sterile chronic multi-focal osteomyelitis ^31^, pyoderma gangrenosum ^32^, and a patient with affected siblings but who was clinically normal despite sharing the same mutations ^33^ ( Table 1).

**Table 1.** Clinical phenotypes in patients with mutations in *RAG1* or *RAG2*.

Phenotype	Autoimmunity described
Severe combined immunodeficiency (SCID)	No
Omenn syndrome	Yes
“Atypical” SCID	Yes
Combined immunodeficiency	Yes
Combined immunodeficiency with granulomas	Yes
Common variable immunodeficiency	Yes
Miscellaneous autoimmunity	Yes
Chronic multi-focal osteomyelitis	No
Pyoderma gangrenosum	No
Idiopathic CD4+ lymphocytopenia	No
No clinical abnormality	No

The most striking feature about these newly described presentations is that they are not new disease manifestations but rather previously described diseases, which now, in some cases, are associated with a defect in *RAG1* or *RAG2*. In some populations, the frequency of individuals carrying mutations in *RAG1* or *RAG2* is estimated to be much higher than is accounted for by the number of SCID or Omenn syndrome patients ^28^, suggesting that many more older patients with less-severe clinical manifestations, including combined immunodeficiency, autoimmune cytopenias, organ-specific autoimmune disease, and antibody deficiency, are yet to be identified. The future challenge will be to decide which patients with these common manifestations should be screened for *RAG* defects and whether a positive finding would alter management of the disease.

How is it that mutations in two small genes can give rise to a plethora of clinical phenotypes? Recent discoveries into the biological effects of hypomorphic mutations on lymphocyte development, in conjunction with modifying environmental factors, help explain the diversity of presentation.

## Thymopoiesis and thymic formation

T- and B-lymphocytes, key cells of the adaptive immune response, are generated in the thymus and bone marrow, respectively. Each naive lymphocyte has a unique antigen receptor and lymphocyte receptor diversity, generated by stochastic genetic rearrangements that cut, splice, and modify lymphocyte receptor variable region gene segments at the lymphocyte receptor loci, that is estimated to be in the region of 10 ^20^ α–β chain combinations for T-lymphocyte receptors ^34^ or heavy-light chain combinations for B-lymphocyte receptors.

DNA double-strand breaks introduced by an endonuclease complex coded by *RAG1* and *RAG2*, which randomly incise DNA at highly conserved sequences of DNA (recombination signal sequences) that flank all variable (V), diversity (D), and joining (J) coding regions, initiate T-lymphocyte receptor gene segment re-arrangement in the thymic cortex, enabling the assembly of interspersed V(D)J gene elements, a process known as V(D)J recombination.

The stochastic re-assembly of V(D)J gene segments leads to the formation of a wide receptor repertoire. The random nature of receptor formation inevitably generates some strongly self-reactive lymphocyte receptors, which carry the risk of initiating autoimmune responses. Developing thymocytes that successfully re-arrange the antigen receptor therefore navigate a rigorous intra-thymic two-stage selection process to identify and remove these potentially damaging self-reactive T-lymphocytes ^35^. In the first phase, known as positive selection, thymocytes are exposed to a self-peptide/major histocompatibility complex (MHC) presented by cortical thymic epithelial cells in the thymic cortex. By recognition of this complex with intermediate receptor affinity, thymocytes can advance to the next developmental phase, ensuring that antigen is recognized in the company of self-MHC molecules. Receptors that fail to bind the complex or bind with high affinity facilitate apoptosis of the thymocyte.

Surviving thymocytes migrate to the thymic medulla and submit to the second stage of thymocyte selection, designated negative selection, during which the antigen receptor interacts with a self-peptide/MHC complex presented by medullary thymic epithelial cells and dendritic cells. Medullary thymic epithelial cells express a wide range of ectopic peripheral tissue-restricted self-antigens (TRA), partly controlled by the Autoimmune Regulator (AIRE) and Fezf2 transcription factors ^36– 38^, exposing a “molecular mirror of peripheral self”. Antigen receptors that bind with high affinity to the TRA/MHC complexes are deleted, or forced down a regulatory T-lymphocyte developmental pathway, as otherwise these have the potential to elicit autoimmunity ^39^. Negative selection is an indispensable function of central tolerance, a key process that eliminates auto-reactive T-lymphocytes.

A calm and uninterrupted thymic microenvironment is required for normal T-lymphocyte development ^40^; however, typical thymic architectural development relies on “thymic crosstalk”, i.e. feedback from the developing thymocytes ^41^. CD4+ thymocytes express CD40 ligand and RANK ligand, which provide signals to medullary thymic epithelial cells ^42^, enabling cellular maturation and subsequent expression of *AIRE*.

## Effect of *RAG* deficiency on thymic development

Seminal studies in infants with genetic defects that severely impair T-lymphocyte development have shown disrupted thymic architecture, with diminished thymic epithelial cell development, absent AIRE expression, and severely reduced FOXP3+ regulatory T-lymphocytes not seen in mutations that permit T-lymphocyte development ^43^. Furthermore, disturbed AIRE expression with diminished self-antigen expression has been demonstrated in the thymus of infants with hypomorphic *RAG* mutations ^44^. These findings demonstrate that significant impairment of T-lymphocyte development may diminish central and peripheral tolerance mechanisms, increasing the risk of escape of highly autoreactive T-lymphocyte clones and thus the risk of developing autoimmunity for an affected individual. Nonsense mutations in *RAG1* or *RAG2* disrupt the function of RAG endonucleases profoundly and result in complete failure to initiate antigen receptor V(D)J re-arrangement, which causes failure of progression of T- and B-lymphocyte progenitors beyond the DN3 and pre-B-1 stage of development. This gives rise to a T-B-NK+ SCID phenotype. Thymic development is interrupted, and no T-lymphocytes are able to develop because of the intrinsic hematopoietic stem cell defect. In hypomorphic *RAG* deficiency, residual V(D)J function is permissive of some T-lymphocyte development, and so thymic development occurs, albeit in an aberrant fashion.

## Role of *RAG* deficiency on T-lymphocyte-mediated clinical features

Increasing evidence demonstrates advancing restriction in the *IGH* and *TRB* repertoire associated with increased T- and B-lymphocyte clonality in patients with associated increasingly severe RAG deficiency, step-wise from normal through combined immunodeficiency with granulomas/autoimmunity, leaky SCID, and Omenn syndrome ^45^. A reduction in TCRβ diversity accompanied by distorted use of V, D, and J gene segments and restriction in CDR3 length diversity are more pronounced in patients with a more-severe clinical phenotype ^45^. This repertoire restriction is determined by the residual endonuclease activity conferred by the mutation in *RAG1* ^46^ or *RAG2* ^47^, which permits some V(D)J recombination in less-severe mutations and permits the formation of a few T-lymphocyte clones which experience impaired positive and negative selection as they pass through the disrupted thymic structure.

However, more recent findings also directly implicate the effect of the *RAG* mutation on V(D)J recombination, preferentially conferring an autoreactive phenotype on the lymphocyte, leading to subsequent development of dysregulated immune responses, as well as the impact of the impaired central and peripheral tolerance mechanisms. Hypomorphic *RAG* mutations are more likely to lead to the development of an antigen receptor that is autoreactive as well as reduce the likelihood of deletion through central tolerance because of disrupted thymic architecture.

High-throughput sequencing of the T-lymphocyte receptor from patients with *RAG* mutations has demonstrated less junctional diversity and smaller CDR3 regions than seen in patients with Omenn syndrome caused by other gene mutations ^48^. During normal lymphocyte development, T-lymphocyte receptor and immunoglobulin gene rearrangements progress through a unique somatic recombination development. The TCRα locus employs a multistep recombination process, progressing from use of proximal TRAV elements to distal TRAV elements. This process is arrested by downregulation of RAG1/2 expression following positive selection of thymocytes. The persistent reduced recombinase activity over successive waves of TCRα rearrangement in patients with RAG defects manifests as a bias in TCRα use towards more proximal TRAV/TRAJ associations. These specific patterns can be detected using flow cytometric methods, which have been observed in patients with V(D)J recombination defects, including RAG deficiency ^49^.

Hypomorphic *Rag* murine models demonstrate impaired gene rearrangement at the *Igh*, *Igk*, and *Trb* loci, which limits antigen receptor diversity from a very early stage in thymocyte development ^50^. Hydrophobic residues within the T-lymphocyte receptor CDR3 region have been associated with the development of autoreactive T-lymphocytes ^51^, and specific cysteine patterns in the same CDR3 region have also been shown to predict the development of autoreactivity ^52^. In the conventional CD4+ T-lymphocytes of patients with combined immunodeficiency or atypical SCID, associated with hypomorphic *RAG* mutations, enhancement for hydrophobic amino acids associated with auto-reactivity and a reduction in those hydrophilic amino acids that limit autoimmunity have been identified in the CDR3 region ^53^. This may reveal an intrinsic bias favoring the production of auto-reactive T-lymphocytes as well as impaired negative selection due to impaired central tolerance, indicated by reduced expression of AIRE ^44^.

Additionally, there is a reduction in the number ^43, 44, 50, 54^ and function ^53, 55^ of regulatory T lymphocytes in these patients, which will have an impact on peripheral tolerance.

## Role of *RAG* deficiency on B-lymphocyte-mediated clinical features

As well as a raised IgE, IgM and IgG autoantibodies have been detected in patients with hypomorphic *RAG* mutations, including those with Omenn syndrome, and especially those with autoimmune manifestations with combined immunodeficiency or granulomas, against a wide range of auto-antigens ^56^. Amongst these autoantibodies are neutralizing anti-cytokine antibodies directed against anti-IFN-α or anti-IFN-ω, which may be precipitated by environmental triggers such as viral infection ^28, 57^. Murine models with hypomorphic *Rag* mutations produce high levels of low-affinity autoantibodies, associated with impaired receptor editing and an increase in serum B-lymphocyte-activating factor (BAFF) concentrations, also identified in patients with Omenn syndrome and leaky SCID, implicating B-lymphocytes in the autoreactivity found in these patients ^57^. BAFF is important in promoting B-lymphocyte survival. Reduced levels of BAFF receptor are expressed by anergic self-reactive B-lymphocytes compared to naïve B-lymphocytes ^58^, and in normal homeostasis, auto-reactive cells are therefore eliminated from the pool of circulating naïve B-lymphocytes. RAG-mutated patients and mouse models exhibit profound B-lymphocytopenia, and the higher circulating levels of BAFF enable the survival of immature auto-reactive B-lymphocytes ^56, 59^. These patients have more class-switched immunoglobulin heavy chain transcripts and an increased rate of somatic hypermutation, indicating *in vivo* B lymphocyte activation ^45, 57^.

## Role of *RAG* deficiency on natural killer cell-mediated clinical features

Invariant NK T-lymphocyte populations protect against autoimmunity and graft-versus-host disease ^60, 61^. Development requires thymic-dependent, RAG-initiated V(D)J recombination. These cells are absent in patients with Omenn syndrome, the lack of which may contribute to the autoimmune phenomena seen in these patients ^62^.

Conventional NK cells are found in normal or sometimes increased numbers in patients with RAG mutations, regardless of the clinical presentation. Within the NK population, however, is a higher-than-normal proportion of immature (CD56 ^bright^) NK cells, which exhibit both increased perforin content and enhanced degranulation activity ^63^. This heightened NK cell-mediated cytotoxicity may be responsible for the increased rate of graft rejection observed after hematopoietic stem cell transplantation (HSCT) in patients with RAG-deficient SCID ^64^, in contrast to patients with other NK+ SCID phenotypes, such as IL7Ra deficiency. An increased cytotoxic reaction has been observed also in NK lymphocytes from Rag ^–/–^ mice, although the NK cells display a mature and activated phenotype in contrast to patients ^65^.

## Role of the environment in clinical manifestations of *RAG* deficiency

The degree of residual protein function clearly plays a role in determining clinical phenotype, but it is not the sole determinant, because patients who share similar *RAG* mutations, which confer similar immunobiological effects, may present with different clinical phenotypes ^66^. Omenn syndrome can evolve from SCID over a period of weeks, as internal or external antigens, including viruses ^14^, drive T-lymphocyte clonal expansion. Anti-cytokine antibodies, such as anti-IFN-α or anti-IFN-ω, seen in patients with *RAG*-mutated combined immunodeficiency or atypical SCID, may be produced as a result of viral infection in predisposed patients ^57, 67^. The intestinal microbiota may also influence the development of autoimmunity. In murine models of RAG deficiency, inflammatory bowel disease mediated by intestinal CD4 T _H_1/T _H_17 T-lymphocytes has been ameliorated by manipulating the intestinal flora, resulting in the reduction of gut tropic T-lymphocytes and reduced IgE levels ^68^ ( Table 2).

**Table 2.** Mechanisms of autoimmunity in RAG deficiency.

Lymphocyte population	Mechanism of autoimmunity
T-lymphocyte receptor development	Restriction in T cell receptor (TCR) repertoire
	Reduction in TCRβ diversity
	Distorted use of V, D, and J gene segments
	Restriction in CDR3 length diversity
	Reduction in VDJ junctional diversity
	Bias in TCRα use towards more proximal TRAV/TRAJ associations
	Enhanced use of hydrophobic amino acids in the CDR3 region
	
T-lymphocytes – central tolerance	Impaired T-lymphocyte development leading to disordered thymic architecture
	Impaired positive and negative thymocyte selection
	Failure of apoptosis of autoreactive T-lymphocytes due to diminished Autoimmune Regulator (AIRE) expression
T-lymphocytes – peripheral tolerance	Diminished regulatory T-lymphocyte numbers
	Impaired regulatory T-lymphocyte function
	
B-lymphocyte receptor development	Distorted use of V, D, and J gene segments
	Reduction in antigen receptor diversity
	B-lymphocyte-activating factor (BAFF)-associated survival of immature auto-reactive B-lymphocytes
	Production of auto-antibodies
	Production of neutralizing anti-cytokine antibodies
	
Environmental factors	Viral-driven clonal T-lymphocyte expansion
	Viral-driven clonal T-lymphocyte anti-cytokine antibody production
	Intestinal microbiota-driven expansion of gut-trophic T-lymphocytes

## Therapy

For the most-severe disease manifestations, including SCID, atypical SCID, and Omenn syndrome, HSCT is the standard of care. Initial management should be directed at the treatment of pre-existing infections, appropriate antimicrobial prophylaxis, and immunoglobulin replacement. Nutritional support with hyper alimentation or total parenteral nutrition is often required. Skin care is critical, and topical treatments should be utilized, including steroid ointments and topical tacrolimus, as well as topical anti-bacterial washes and emollients and barrier creams. Patients frequently experience super-added bacterial infection; *Staphylococcus aureus* is usually implicated. These infections should be treated aggressively. Calcineurin inhibitors and other systemic immunosuppression may improve the inflammation ^69^, which may affect any and all organs. Serotherapy may be required to remove auto-inflammatory cells, although immunosuppression in the presence of active infection can present specific challenges. Careful pre-transplant care is critical to a successful outcome following HSCT. In the modern era, survival from HSCT for primary immunodeficiencies is similar whether a matched sibling, matched unrelated, or mismatched T-lymphocyte-depleted donor is utilized. The outcome of HSCT for RAG deficiency (characterized as T-B- SCID) has been significantly worse than other forms of SCID ^70^, but a recent report has shown similar survival rates for RAG-SCID as for other genetically defined T-B+ SCID phenotypes ^71^. Thymic and bone marrow niches are occupied by developmentally arrested pre-cursors in RAG-deficient patients, and so long-term immune reconstitution is likely to be achieved only after the use of a conditioning regimen ^72^, preferentially with serotherapy which targets NK cells. Graft failure is common in RAG deficiency ^64, 71^, perhaps partly mediated by highly reactive NK cells which exhibit enhanced degranulation and increased amounts of perforin ^63^. The best results are achieved if patients are infection-free at the time of transplantation ^71, 73^, most likely accomplished if they are detected by newborn screening ^74^.

Genetic therapy using gene addition is likely to be the next therapeutic advance in treating these patients. Autologous stem cells are used, removing the risk of graft-versus-host disease and rejection. Gene therapy for X-linked SCID and for adenosine deaminase-deficient SCID has been established, with a licensed vector for adenosine deaminase-deficient SCID available in Europe. Survival results are excellent, and the lentiviral vectors have a better safety profile than gammaretroviral vectors ^75, 76^. Preclinical lentiviral vector gene therapy studies for RAG1 and RAG2 deficiency using animal models have shown some success ^77, 78^ to date without the risks of oncogenesis described when older retroviral vectors were used ^79^; clinical trials using lentiviral vectors to treat patients are now in progress ^80^. The very best outcomes may be possible when patients are treated infection-free as newborns using a gene therapy protocol with chemotherapy-free antibody-based conditioning regimens ^81, 82^.

## Conclusion

It is now clear that hypomorphic mutations in *RAG* genes can result in protean manifestations of immunodeficiency, immunodysregulation, or autoimmunity, even manifesting in late adolescence or early adulthood. New understandings of the biological effects of these mutations, particularly on the development of T- and B-lymphocyte receptors, help explain these clinical presentations. For the more-severe manifestations, emerging gene therapy technologies may offer novel therapy options.
